# Cockayne syndrome without UV-sensitivity in Vietnamese siblings with novel *ERCC8* variants

**DOI:** 10.18632/aging.204139

**Published:** 2022-06-22

**Authors:** Nguyen Thuy Duong, Tran Huu Dinh, Britta S. Möhl, Stefan Hintze, Do Hai Quynh, Duong Thi Thu Ha, Ngo Diem Ngoc, Vu Chi Dung, Noriko Miyake, Nong Van Hai, Naomichi Matsumoto, Peter Meinke

**Affiliations:** 1 Institute of Genome Research, Vietnam Academy of Science and Technology, Hanoi, Vietnam; 2 Institute of Virology, School of Medicine, Technical University of Munich/Helmholtz Zentrum München, Munich, Germany; 3 Friedrich-Baur-Institute, Department of Neurology, LMU Klinikum, Ludwig-Maximilians-University Munich, Munich, Germany; 4 Vietnam National Children’s Hospital, Hanoi, Vietnam; 5 Department of Human Genetics, Yokohama City University Graduate School of Medicine, Kanagawa, Japan; 6 Department of Human Genetics, Research Institute, National Center for Global Health and Medicine, Tokyo, Japan

**Keywords:** Cockayne syndrome, DNA excision repair, ERCC8, segmental progeroid disease

## Abstract

Cockayne syndrome (CS) is a rare progeroid disorder characterized by growth failure, microcephaly, photosensitivity, and premature aging, mainly arising from biallelic *ERCC8* (CS-A) or *ERCC6* (CS-B) variants. In this study we describe siblings suffering from classical Cockayne syndrome but without photosensitivity, which delayed a clinical diagnosis for 16 years. By whole-exome sequencing we identified the two novel compound heterozygous *ERCC8* variants c.370_371del (p.L124Efs*15) and c.484G>C (p.G162R). The causality of the *ERCC8* variants, of which one results in a frameshift and the other affects the WD3 domain, was tested and confirmed by a rescue experiment investigating DNA repair in H_2_O_2_ treated patient fibroblasts. Structural modeling of the p.G162R variant indicates effects on protein-protein interaction. This case shows the importance to test for *ERCC6* and *ERCC8* variants even if patients do not present with a complete CS phenotype.

## INTRODUCTION

Cockayne syndrome (CS) is a rare autosomal recessive disease characterized by growth failure, microcephaly, and progeroid appearance [1]. Additional clinical features include cold hands and feet, bilateral hearing loss, photosensitivity, tremors, joint contractures, progressive loss of body fat, and cataracts [2, 3]. Based on the age of onset and the severity of clinical symptoms, CS can be classified into the types I-III. Type I is a classical or moderate form of Cockayne syndrome with normal prenatal growth, clinical manifestations within the first two years of age, and an average lifespan of 16 years. Type II is a severe or early-onset form with abnormalities at birth, little or no postnatal neurological development, and a maximum lifespan of seven years. Patients with CS type III, a mild or late-onset form, have an average lifespan of 40 to 50 years.

Recessive variants in excision repair cross-complementation group 8 (*ERCC8*; MIM #216400; CS-A) and 6 (*ERCC6*; MIM #133540; CS-B) genes have been described to be causative for CS [4]. *ERCC8* encodes the DNA excision repair protein ERCC-8 (CSA), a 396-amino-acid protein, comprising 7 WD (tryptophan-aspartic acid dipeptide) domains [5, 6]. *ERCC6* encodes DNA excision repair protein ERCC-6 (CSB), a 1493-amino-acid protein which is a member of the SNF2/SW12 ATPases family. CSB has a central ATPase domain (residues 510 - 960) containing seven conserved helicase motifs, which support its role in chromatin remodeling, transcriptional regulation, and DNA repair [7, 8]. CS proteins play important roles in the transcription-coupled sub-pathway of nucleotide excision repair (TC-NER) for UV-induced DNA damage [9, 10]. Therefore, pathogenic variants in CSA and CSB result in the deficit of TC-NER and subsequently cause various hereditary diseases, including CS [11]. In total, about 70 % of the described CS cases are caused by *ERCC6* variants and 30 % by *ERCC8* variants [6].

In this report, we present the first molecular study of a Vietnamese family with CS type I. The patients showed a wide range of clinical manifestations but notably lacked photosensitivity, leading to the need for comprehensive genetic testing.

## RESULTS

### Clinical presentation

**Figure 1:**
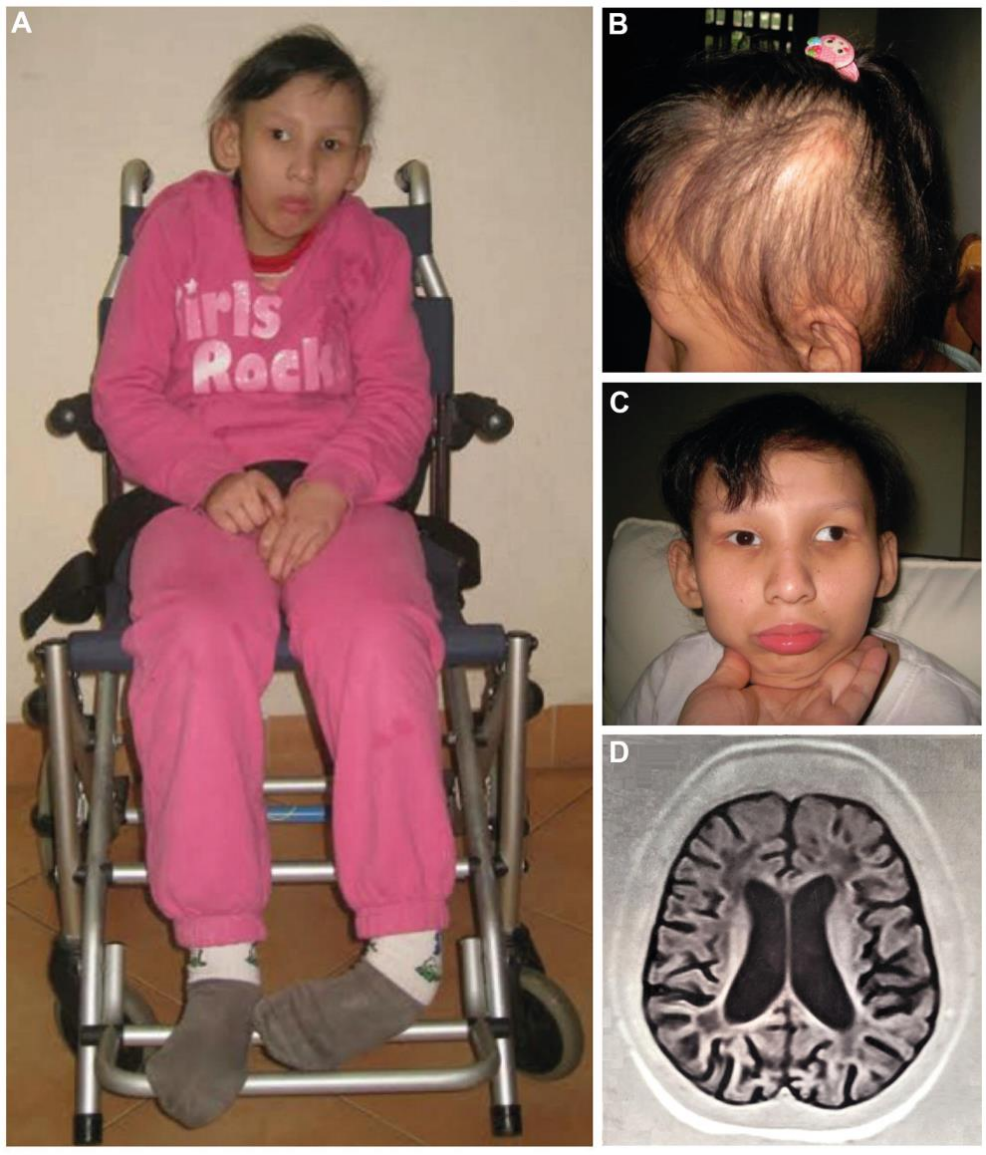
Proband at the age of 16 (**A**–**C**): (**A**) Full length figure of the proband, thinning hair (**B**) and strabismus at front vision (**C**) were observed. Brain T1-weighted MRI image at the age of 14 years (**D**).

The proband (II-4, Figure 1), one of two twin sisters, was 16 years old at the latest evaluation. Her parents were unaffected but had three miscarriages before she was born. Her twin sister (II-5) and her younger sister (II-6) were also affected. The 4^th^ child (II-7), a boy, was not showing any symptoms at age of 3 years. The proband was born after 39 weeks of gestation as the younger twin of two sisters. The delivery process was elongated due to improper position, and cesarean section was applied. Her birth weight was 2.7 kg (normal range: 3.2 ± 0.45). Abnormal facial appearance with sunken orbits, small eyes, snub and small nose, narrow mouth, clutching hands, microcephaly, finger-cross were noticed at birth.

She suffered pneumonia on the 15^th^ day and had been treated in the Vietnam National Children’s Hospital for two weeks. Postnatal feeding difficulties due to the small mouth of the patient were noted. She drank a small amount of milk compared with children of the same age, vomited 3 to 5 times per day, and had sleep problems. Signs of hunger and tear were rarely observed. Her growth failure was noticed at 12 months with a weight of 8.3 kg (normal range: 8.9 ± 1.2) and height of 67 cm (74 ± 2.5). Motor development was slow with incapability of standing at 12 months. She was able to stand and walk at 2 - 3 steps without proper balance at 27 months, although she underwent physiotherapy from the 18^th^ month of life. At the age of 31 months, she was assessed by the Denver developmental screening test to identify overall development. The results indicated that her developmental quotient (DQ) was 60-65 with personal-social capability, fine motor-adaptive behavior, and language development of an 18-month-old child and gross motor skill of a 14-month-old child.

She could understand simple things and speak a few words but not whole sentences. Moreover, she lacked the capability of learning, focusing, and logical thinking. At the age of eight, she showed regression in moving, walking, and recognition. Also aging symptoms appeared more clearly with difficulties in psychomotor skills; strabismus started to develop along with decreased vision, and limb muscles weakened over time. Around 12 years of age, her face showed obvious signs of premature aging.

At the age of 14, her body weight and height were 27 kg (normal range: 49.7 ± 10.2) and 118 cm (normal range: 160.6 ± 6.2), respectively. She caught a left-foot contracture, making her wheelchair-bound (Figure 1A). As she got older, her hair became sparser, thinner, and drier (Figure 1B). No dermal photosensitivity was observed. She developed strong strabismus (Figure 1C).

Magnetic resonance imaging (MRI) scan of the brain at the age of 14 showed brain atrophy, particularly atrophy in the cerebellum and ventriculomegaly (Figure 1D). Moreover, the abdominal ultrasound revealed atrophic kidneys with the right and left kidneys were 58 mm and 62 mm long, respectively (normal range: 100.2 ± 7.5). A dramatic increase in creatinine of 218 μmol/L (normal range: 44.00 – 88.00 μmol/L) and urea of 11.8 mmol/L (normal range: 1.70 – 8.30 mmol/L) also suggested renal failure in the patient. Consequently, the patient deceased at age of 18 due to multi organ failure (including renal and liver failure, respiratory failure, and brain atrophy). The main clinical manifestations of the proband and her two affected sisters as well as a comparison to other published cases are summarized in Table 1.

**Table 1:** Major clinical features of the index patient and her two affected sisters in comparison with other studies.

**Patient ID**	**Current study**			**Other studies**		
	**II-4**	**II-5**	**II-6**	**(Nance and Berry, 1992) [1]**	**(Natale, 2011) [12]**	**(Wilson et al., 2016) [2]**
Gender	Female	Female	Female	60/140	n/a	n/a
Gestational age	39 weeks	39 weeks	38 weeks	n/a	n/a	n/a
Delivery	Caesarean section	Caesarean section	Caesarean section	n/a	n/a	24/90
Birth weight (3.2±0.45)	2.7 kg	2.4 kg	3.3 kg	n/a	<5^th^ percentile	Between 2^nd^ and 91^st^ percentiles
Age at evaluation	22 months	22 months	At birth	n/a	n/a	n/a
Growth failure	Yes	Yes	Yes	140/140	45/45	90/90
Microcephaly	43.2 cm (<3^rd^ percentile)	42.1 cm (<3^rd^ percentile)	42.7 cm (<3^rd^ percentile)	n/a	n/a	n/a
Wrinkled face	Yes	Yes	Yes	n/a	n/a	n/a
Strabismus	Severe	Severe	Severe	10/128	n/a	n/a
Cataract	No	No	No	46/128	24/45	49/102
Psychomotor delay	Yes	Yes	Yes	50/131	45/45	n/a
Intellectual disability	Severe	Severe	Severe	5/131	45/45	n/a
Speech abnormalities	Yes	Yes	Yes	28/131	45/45	n/a
Peripheral coldness	Yes	Yes	Yes	n/a	45/45	90/102
Tremor	Yes	Yes	Yes	42/131	36/45	67/102
Sensorineural hearing loss	Mild (41- 70 dB)	Mild (41- 70 dB)	Mild (41- 70 dB)	47/78	43/45	76/102
Feeding difficulty	Yes	Yes	Yes	11/131	24/45	49/102
Gastroesophageal reflux	Yes	Yes	Yes	n/a	27/45	58/102
Joint contractures	Yes	No	Yes	n/a	45/45	65/102
Muscle weakness	Yes	Yes	Yes	n/a	n/a	80/102
Photosensitivity skin	No	No	No	67/92	45/45	76/102
Dry skin	Yes	Yes	Yes	n/a	n/a	n/a
Thin and dry hair	Yes	Yes	Yes	n/a	n/a	47/102
Dental caries	Yes	Yes	Yes	43/50	26/45	47/102
Renal failure	Yes	No	Yes	2/140	n/a	n/a
Menstrual cycles	Irregular	Irregular	Irregular	n/a	31/31	n/a

n/a, not available.

### Genetic analysis

**Figure 2:**
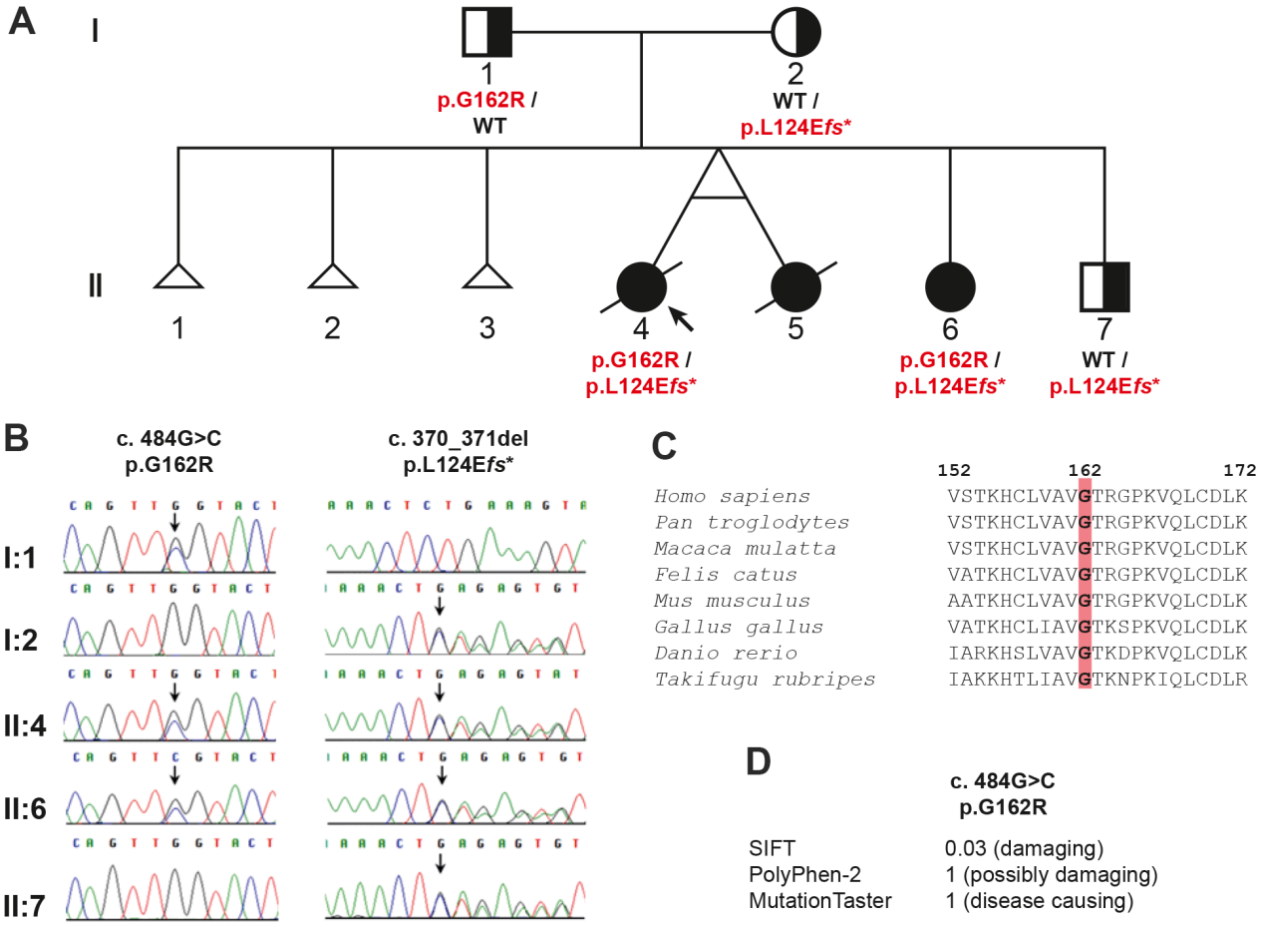
Pedigree analysis of the family in the study: (**A**) the pedigree showing the inheritance of the *ERCC8* variants, (**B**) chromatograms for the variants verified by Sanger sequencing, (**C**) evolutionary conservation of ERCC8 p.G162, and (**D**) prediction of the effect of the missense variant.

Whole exome sequencing (WES) was used for genetic testing of the proband. After filtering out variants with a minor allele frequency > 0.01 in the 1000 Genome Project or ExAC, a total of 796 variants remained within the coding region and adjacent intronic region within 30 bps from exon-intron borders. The three affected siblings would indicate the autosomal recessive inheritance of their condition. Searching for variants fitting with autosomal recessive inheritance 71 variants in 29 genes remained as potential causative changes (Supplementary Table 1). Based on the function of the gene products, the two heterozygous variants NM_000082.4: c.484G>C (p.G162R) and c.370_371del (p.L124Efs*15) in the *ERCC8* gene were the most promising candidates. Both variants were confirmed in the proband (II-4) and her affected sister (II-6) by Sanger sequencing (Figure 2A, 2B). The father (I-1) was heterozygous for c.484G>C, while the mother (I-2) and the younger brother (II-7) were heterozygous for c.370_371del (Figure 2A, 2B). Furthermore, these two variants were not found in 192 unrelated blood donors of the same ethnic background (Vietnamese).

Both variants were not reported in the exome aggregation consortium (ExAC) or the genome aggregation database (gnomAD). The amino acid glycine (p.G162) is evolutionarily highly conserved from fish to human (Figure 2C) and the mutation of this amino acid was predicted *in silico* to be deleterious by MutationTaster, PolyPhen2, and SIFT (Figure 2D).

### Functional analysis using patient’s fibroblasts

**Figure 3:**
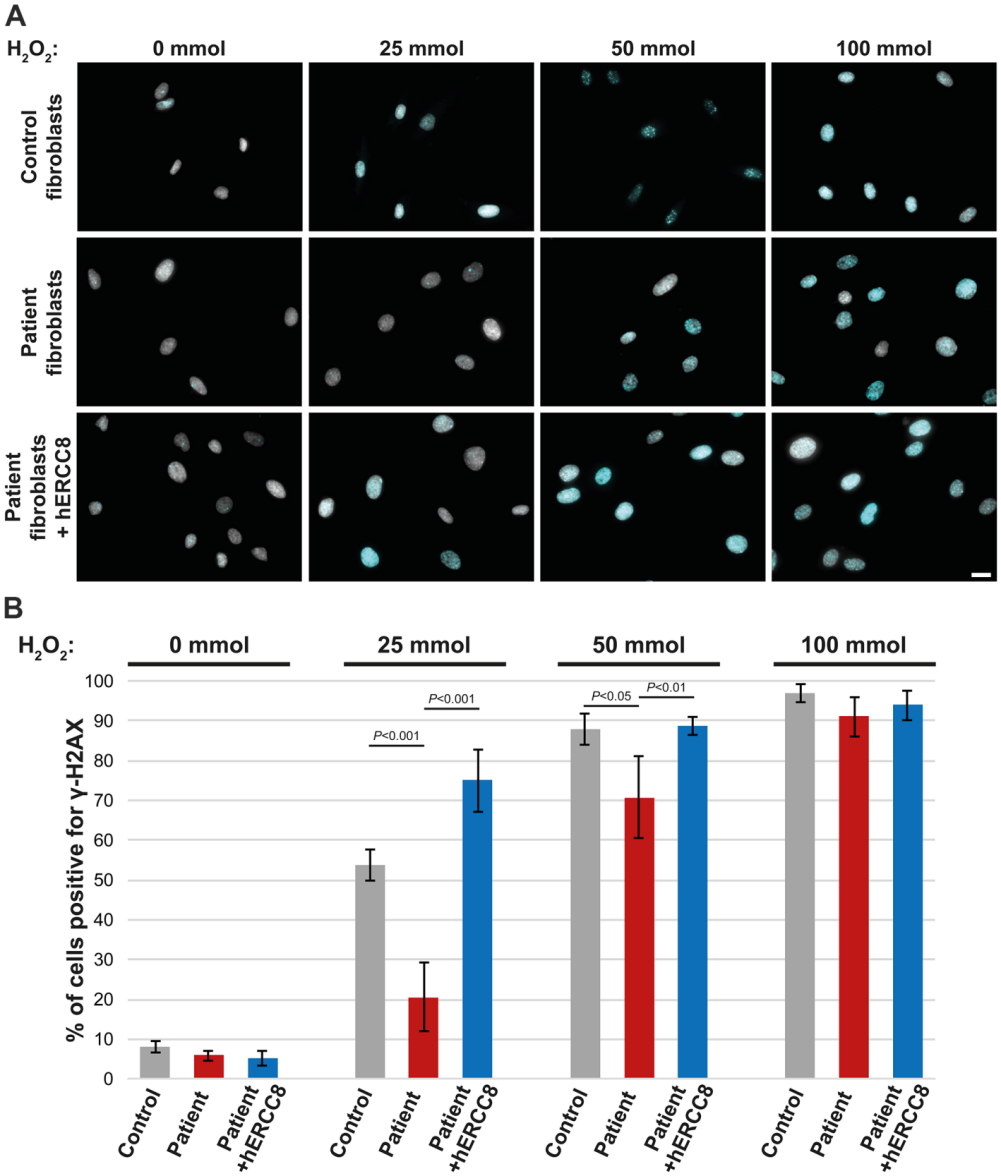
**Rescue experiment: γ-H2AX staining of control, patient, and wt*ERCC8* transduced patient fibroblasts.** (**A**) Cells were stained without as well as following H_2_O_2_ treatment in different concentrations. (**B**) At least three hundred cells per condition from three independent experiments were counted to quantify the number of cells positive for γ-H2AX.

As the *ERCC8* variants we found have not been described to date to be causative for CS, we performed functional analysis using fibroblasts derived from the patient’s skin biopsy. Treatment of control fibroblasts with H_2_O_2_ resulted in a strong accumulation of γ-H2AX in the majority of nuclei depending on the H_2_O_2_ concentration used (Figure 3A, 3B). This was significantly reduced in patient fibroblasts for the lower concentrations (25 and 50 mmol) of H_2_O_2_ (Figure 3A, 3B). To investigate the causality of the *ERCC8* variants patient fibroblasts were transduced with wild-type *ERCC8* cDNA using a lentivirus with a selection marker. Transduced patient’s fibroblasts did show similar γ-H2AX accumulation comparable to that of control fibroblasts (Figure 3A, 3B).

**Figure 4:**
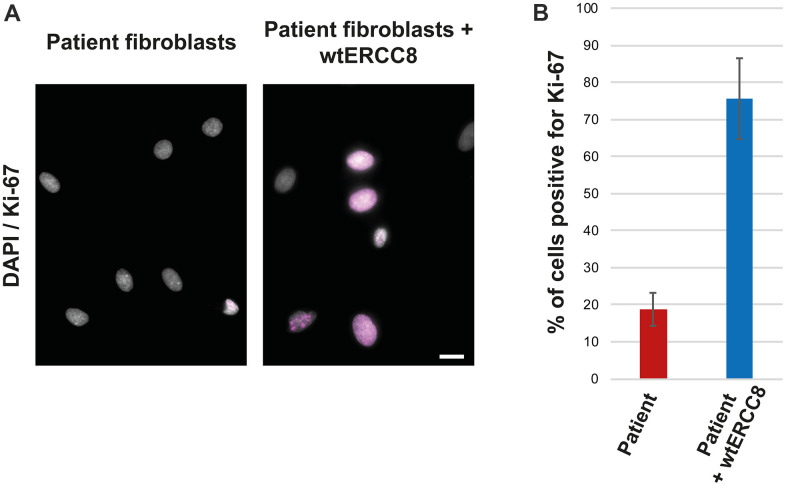
**Rescue experiment: Ki-67 staining of patient and wt*ERCC8* transfected patient fibroblasts.** (**A**) Untreated and transduced patient cells were stained to asses cell proliferation. (**B**) At least three hundred cells per condition from three independent experiments were counted to quantify the number of cells positive for the proliferation marker Ki-67.

The patient fibroblasts proliferated poorly, only about 20 % of the cells were found to be positive for the proliferation marker Ki-67 (Figure 4A, 4B). The transduction with wild-type *ERCC8* cDNA resulted in an increased proliferation rate of more than 70 % (Figure 4A, 4B).

### Structural analysis of the p. G162R variant

**Figure 5:**
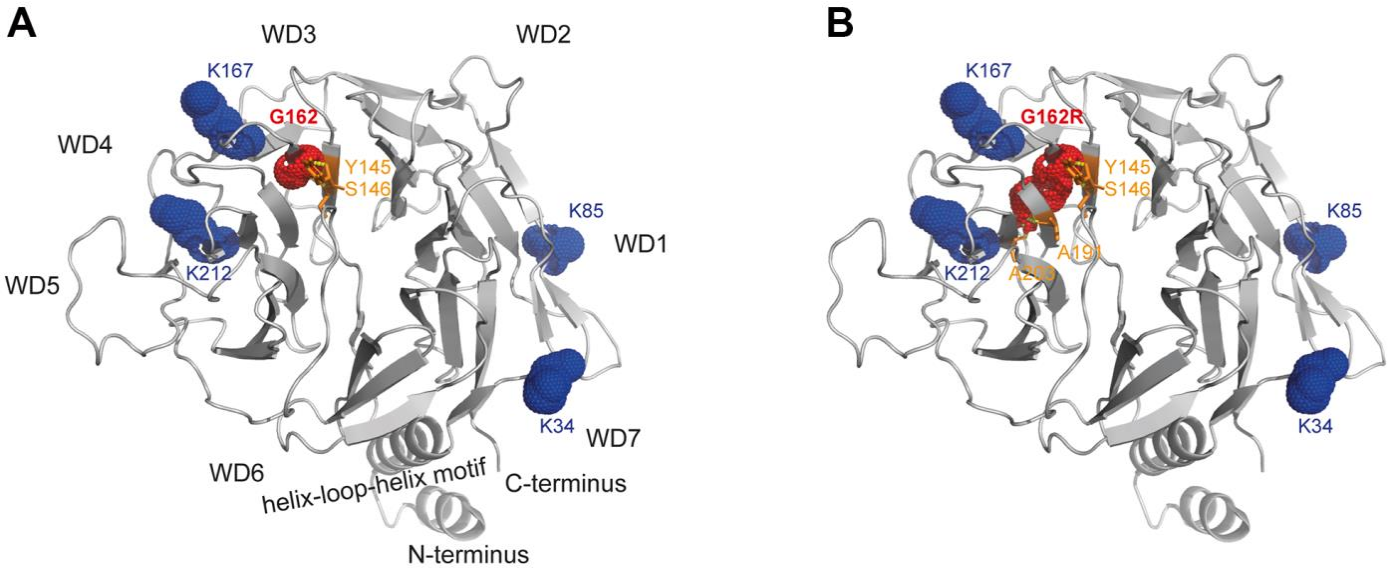
**Structural analysis of WD40-propeller variant p.G162R in CSA.** Structural view of (**A**) WT and (**B**) the p.G162R variant. The CSA structure comprising the seven-bladed WD40 propeller and the helix-loop-helix motif is shown as a ribbon diagram, with p.G162R highlighted in red dots and interacting amino acids in orange sticks, as well as the amino acids relevant for TRiC-association in blue dots. The structural view of CSA (PDB entry 4A11) [13] was generated using the PyMOL molecular graphics system, version 2.4, Schrödinger, LLC.

The CSA-DDB1 complex has been crystallized [13] and shown to interact with the T-complex protein Ring Complex (TRiC) [14]. The glycine at position 162 is located within a ß-sheet being part of the WD-3 blade of the WD40 propeller of the CSA protein. Modeling of the p.G162R variant into this crystal structure showed that arginine results in bonding with additional amino acids (A191/A203) of close-by ß-sheets. But moreover, exchanging the small glycine side chain with the bulky arginine most likely interrupts the functionality of the amino acids K167 and K212, which are essential for TRiC-binding (Figure 5).

## DISCUSSION

In the current study, we describe patients initially diagnosed with an unknown progeroid disease. The clinical characteristics do fit well within the spectrum of CS type I with moderate severity based on the description of Natale [12], except for (i) intellectual disability, which affected all three siblings reported here, but was reported only in very few CS patients before; and (ii) dermal photosensitivity, which is the second most commonly reported single trait in CS patients [1], but was absent in the affected siblings described here. Despite this fitting phenotype, the diagnosis “Cockayne syndrome” was only made after the identification of biallelic *ERCC8* variants, mainly due to the lack of photosensitivity. The whole process took 16 years.

Historically, the diagnosis of CS has been based on the lack of recovery of RNA transcription after UV light-induced DNA damage [15, 16], whereas RNA synthesis recovers very rapidly in normal cells [17]. The presence of cutaneous photosensitivity is therefore a major factor for the diagnoses of CS. Indeed, it has been listed as top-scoring symptom for a recently proposed diagnostic and severity scoring system in CS [18]. Despite this there have been a few reports of CS patients without cutaneous photosensitivity, although mostly reported for CS-B patients [4, 19]. For CS-A, we could find 7 cases without cutaneous photosensitivity [19]. Thus, it appears necessary and more effective to conduct molecular testing for CS even if key clinical features like photosensitivity are missing.

According to the public database of HGMD professional, 78 variants on *ERCC8* have been reported in patients with CS-A [4, 19–27]. In the patients reported here we identified a combination of compound heterozygous frameshift and missense variants which had not been described previously. The frameshift variant (p.L124Efs*15) located in the WD-2 is predicted to result in a truncated polypeptide, but could also result in no protein expression at all from that allele. Like this variant, most of the CS-causing variants resulted in a truncated and/or non-functional CSA protein or loss of CSA. Contrary, for most of the missense variants an explanation is missing. Modeling of the p.G162R variant into the crystal structure indicates effects on the interaction of CSA with other proteins. Based on the CSA-DDB1 crystal structure [13], we found that introducing the bulky side chain of arginine at position 162 hits one of the motifs (K167/K212) that are essential for TRiC binding [14]. Therefore, we propose that the mutated bulky side chain of arginine within the binding region to TRiC is likely to interfere with the TRiC-mediated folding and stabilization of CSA [14]. Thus, it is possible that only the CSA allele containing the p.G162R variant results either in a reduced amount of properly folded and thus functional protein or that a misfolded CSA has different interactions and therefore partially different functionalities.

The CSA protein is involved in several cellular functions including DNA repair, transcription, oxidative stress response and protecting cells from senescence [28]. Especially its function in the transcription-coupled nucleotide excision repair (TC-NER) of damaged DNA is well investigated: CSA is a member of the E3 ubiquitin ligase complex, which is responsible for the ubiquitination and degradation of TC-NER proteins when the repair is finalized [29]. But CSA has also been shown to be involved in the repair of DNA double strand breaks (DSBs) by non-homologous end joining (NHEJ) [9]. The finding that patients carrying homozygous CSA p.W361C variants suffer from UV-sensitive syndrome without any features of premature aging [30] could indicate that the CSA functions in TC-NER and NHEJ are separate from each other and different parts of the protein are responsible for the respective function.

To investigate if the *ERCC8* variants are causative for the patient phenotype we did induce DNA damage in patient fibroblasts using hydrogen peroxide. Hydrogen peroxide has been shown to induce DNA breaks as well as DNA lesions [31]. We found reduced γ-H2AX accumulation (a marker for DNA repair at DSBs [32]) in patient cells following the H_2_O_2_ treatment. A rescue experiment, in which we transduced wild-type *ERCC8* into the patient fibroblasts, did show a restoration of γ-H2AX accumulation following DNA damage. Thus we conclude that the compound heterozygous CSA variants p.L124Efs* and p.G162R are causative for CS. This also further supports the idea of a specific CSA function in DSB repair.

Furthermore, the rescue experiment resulted in an increased proliferation rate, restoring the very poor proliferation observed in the patient cells. This further supports the causality of the identified *ERCC8* variants in the described progeroid patients.

In summary, this is the first molecular study of CS in Vietnam. Our clinical and functional findings strengthen the link between DNA impairment repair and progeroid phenotypes of the patient. The present findings not only enrich the known spectrum of *ERCC8* in CS, but also emphasize the value of WES in clinical diagnosis and identification of disease-causing variants.

## MATERIALS AND METHODS

### Patients materials and ethics

For DNA analysis, EDTA blood collection tubes were used to collect blood from all study participants. Genomic DNA of the index patient, her family members, and unaffected controls was extracted using the QIAamp DNA Blood Mini Kit (Qiagen, Germany). To gain skin fibroblasts a small skin biopsy was taken. All subjects analyzed in this study gave written informed consent before participation. The study was approved by the Institutional Review Board of the Institute of Genome Research, Vietnam Academy of Science and Technology (No: 2-2019/NCHG-HĐĐĐ).

### Whole exome sequencing

The genomic DNA of the index patient was prepared by the Covaris model S2 system (Covaris, Woburn, MA, USA). Genomic DNA was partitioned using the SureSelectXT Human All Exon 50 Mb, v5 libraries (Agilent Technologies, Santa Clara, CA, USA) on a 2500 platform (Illumina, USA) with 101 bp paired-end reads. Quality-controlled reads were aligned to the human reference genome (UCSC hg19, NCBI build 37.1) using Novoalign (http://www.novocraft.com/products/novoalign/). Polymerase chain reaction (PCR) duplications were removed using Picard (http://broadinstitute.github.io/picard/). Variants were called using Genome Analysis ToolKit (GATK) (https://www.broadinstitute.org/gatk/index.php).

### PCR and Sanger sequencing

Variants of the *ERCC8* gene obtained from whole exome sequencing were validated using Sanger sequencing. Additionally, 192 unrelated Vietnamese individuals were analyzed for the identified *ERCC8* variants. The target sites and flanking regions of the gene were amplified using Eppendorf Mastercycler with specific primers. Primer information will be provided if requested. All obtained PCR fragments were treated with ExoSAP-IT, Affymetrix (Thermo Fisher Scientific, USA). The purified PCR products were sequenced on ABI3500 automated sequencer (Applied Biosystems, Waltham, MA, USA) with the same primers used for the PCR. The sequencing results were compared with reference DNA sequences of *ERCC8* published in GenBank with accession number NM_000082.

### Prediction tools

To predict the pathogenicity of the identified *ERCC8* variants, SIFT (https://sift.bii.a-star.edu.sg), Polyphen2 (http://genetics.bwh.harvard.edu/pph2), and MutationTaster (http://www.mutationtaster.org) have been used.

### Construction of the viral construct

Wild-type *ERCC8* cDNA gene was introduced into a lentiviral gene expression vector. To verify successful transduction the plasmid contained additional GFP as well as blasticidin-resistance as selection marker. To avoid interference with the secondary structure of the ERCC8 protein both, *GFP* and *ERCC8*, were in independent open reading frames. Cloning and purification of the plasmid as well as viral packaging were done by VectorBuilder.

### Cell culture and transduction

Patient and control fibroblasts were grown in tissue culture using DMEM supplemented with 10 % FCS and Pen/Strep at 37° C in a 5% CO_2_ incubator. Passage numbers were matched for control and patient cells. The patient fibroblasts where transduced at a multiplicity of infection (MOI) of 3.

### H_2_O_2_ treatment

Fibroblasts were treated with 25 mmol, 50 mmol or 100 mmol H_2_O_2_ for 30 minutes, medium was changed back to normal following the treatment and cells were fixed after 1 hour of recovery time.

### Immunohistochemistry

Fibroblasts were fixed with 4 % PFA. Following primary antibodies were used for staining: γ-H2AX (Cell Signaling #9718S), Ki-67 (Thermo Scientific, RM-9106-S0). All secondary antibodies were Alexa Fluor conjugated and generated in donkey with minimal species cross-reactivity (Invitrogen). DNA was visualized with DAPI (4,6-diamidino-2 phenylindole, dihydrochloride).

### Microscopy and image analysis

All images were obtained using an Olympus IX83 fluorescens microscope with 10x Uplan xApo 0.4 and 40x UplanxApo0.95. Image analysis was performed using ImageJ software. For quantification at least 300 cells have been analyzed per condition.

### Structural analysis of the p.G162R variant

The structural views were generated using the PyMOL Molecular Graphics System, version 2.4 (Schrödinger LLC, New York, NY, USA).

## Supplementary Material

Supplementary Table 1
